# Experimental Evaluation of Geometric and Environmental Effects on Mechanically Fastened Non-Crimp Fabric Composites

**DOI:** 10.3390/polym16192744

**Published:** 2024-09-28

**Authors:** Dong-Uk Kim, Ho-Yun Jang, Hyoung-Seock Seo

**Affiliations:** 1 Maritime, DNV Korea Ltd., Geoje 53261, Republic of Korea; dong.uk.kim@dnv.com; 2 Green-Ship Research Center, Research Institute of Medium & Small Shipbuilding, Busan 46757, Republic of Korea; hyjang@rims.re.kr; 3 Department of Autonomous Vehicle System Engineering, Chungnam National University, Daejeon 34134, Republic of Korea

**Keywords:** non-crimp fabric (NCF) composite material, W/D effect, environmental effect, bearing strength, failure mode

## Abstract

Corresponding to marine environmental regulations is important in shipbuilding and marine industries. The application of lightweight composite materials on ships is an effective approach to reducing the emission of greenhouse gases. The mechanical fastening method is a good candidate to assemble composites and conventional metals. The joint geometric and environmental effects are two important factors in mechanically fastened ship and marine structures. In this study, we evaluated the W/D (hole diameter to width ratio) and environmental effects on the bearing strength and failure mode of a mechanically fastened non-crimp fabric (NCF) composite material. To consider the effect of joint geometry, wherein hole diameters of 5, 6, 8, and 10 mm were machined. Further, by selecting three environmental conditions (UV, saltwater and low temperature), we evaluated environmental effects on bearing strength and failure modes of NCF composite specimens. The bearing strength increased as W/D decreased, and the bearing strength of the specimen exposed to low temperature and UV environments increased, while that of the specimen exposed to saltwater remained the same. From the failure mode analysis, the specimen that was exposed to salt fog showed the same failure mode as the unaged specimen. It was observed that the changes in the transition section and new failure mode in the xenon arc and low-temperature specimens.

## 1. Introduction

Fiber-reinforced composite materials have gained significant attention in recent years because of their potential use as a substitute for conventional metals. They have been widely adopted in diverse industries such as defense and aerospace, as well as the R&D of unmanned vehicles, owing to their high specific strength, good stiffness, superior corrosion, and good wear resistance.

However, changes in industrial trends are not readily accepted and adopted by the shipbuilding industry. The main issues associated with this industry are decarbonization and digitalization. The International Maritime Organization (IMO) has set strict environmental regulations such as the reduction in harmful exhaust gases including CO_2_, SOx, and NOx. A massive improvement in propulsive efficiency as well as R&D efforts in eco-friendly propulsion systems is essential to attain carbon neutrality. The integration of composite materials may contribute to reducing the weight of ships and marine structures. Although lightweight composite materials are actively adopted in small ships such as marine leisure ships and fishing boats, they are rarely used in mid or large-sized ships. In recent years, there has been significant attention on the application of lightweight composite materials with excellent durability to reduce the weight of mid to large-sized ships and cater to current eco-friendly trends in shipbuilding and marine industries [1]. 

The fastening method of structures is an important aspect of the integration of lightweight composite materials into ships and marine structures. Materials such as metal are commonly adopted using a welding method for fastening. However, composite materials cannot be fastened through welding. Composite materials are connected to metal materials in ships and marine structures as dissimilar materials and mechanical fastening is a suitable connection method for maintaining structural strength for a long time. The mechanical fastening of the composite material ensures excellent structural strength and durability in the marine environment, in addition to easy partial maintenance [2]. The non-crimp fabric (NCF) composite material, which is a type of lightweight composite material that is integrated into ships and marine structures, has outstanding mechanical properties with respect to in-plane load [3]. The material possesses excellent durability against bearing load in the form of in-plane load, which is applied by mechanical fastening elements such as bolts or rivets. Compared to unidirectional prepreg tape, NCF has similar mechanical strength and can be fabricated at low costs [4]. In addition, because NCF composite material can increase the size of a single module, the number of joints between structures can be reduced [5]. The use of NCF composite material helps reduce the number of joints, and this further improves the structural strength.

The mechanical fastening of composite materials may decrease the structural strength due to stress concentration, crack assembly, and large strain [6]. Therefore, mechanical analysis must be implemented regarding the hole notch, which is machined for mechanical fastening and the elements that reduce the structural strength. Particularly, the impact of joint geometry on the bearing strength of a mechanically fastened structure must be considered. The major geometrical effects include the hole diameter to width ratio (W/D) and the hole diameter to free end length (E/D) [7,8,9]. 

In addition to the joint geometry effect, the effect of the marine environment is an important design element. Because ships and marine structures are exposed to saltwater and UV during long-term operation, the structural strength of composite material structures may be reduced. Generally, composite materials possess relatively high resistance to saltwater environments compared to metal materials; they do not exhibit strength reduction in early stages [10,11,12]. However, the mechanical strength is reduced as exposure to saltwater environments increases [13,14]. Although UV exposure results in a decrease in structural strength, these changes are insignificant in the initial stages, and strength reduction occurs as the exposure time increases [15,16,17]. It is reported that low-degree volumetric shrinkage and resin hardening are the root causes of the decrease in strength [18,19]. There have been very few studies on the effects of the UV environment on mechanically fastened parts of composite materials [20], and further research is necessary [21].

The operation of ships and marine structures in polar environments has increased considerably in recent years owing to the exploration and development of future polar energy [22]. An ideal design process is one that reflects the changes in mechanical properties and the characteristics of the structural strength of composite materials in polar as well as marine environments. Generally, composite materials tend to be brittle, and their strength and stiffness increase in low-temperature environments [23,24,25]. Some studies have examined the impact of a low-temperature environment on mechanically fastened parts [26,27,28]. According to the study by Walker [28], a low-temperature environment may have a direct impact on the mechanical properties and failure mode of mechanically fastened parts of composite materials. 

Several studies have been conducted on the impact of joint geometry, marine environment, and low-temperature environment on composite materials and the mechanically fastened parts of composite materials. However, to the best of the author’s knowledge, no previous study has examined that considers both the effects of the joint geometry of mechanically fastened parts of NCF composite materials as well as those of marine and low-temperature environments. Therefore, in this study, we examined the effects of joint geometry and marine environments on the mechanical behavior, response, and failure mode of mechanically fastened parts of NCF composite materials. We conducted an experiment on the static bearing strength after exposing the mechanically fastened part of an NCF composite material specimen to marine environments such as saltwater, UV light, and low-temperature environments. 

The aim of this work is to investigate the W/D effect and environmental effects on the mechanical joints of NCF composite materials and to evaluate the dominance of each effect on bearing strength and failure modes. Furthermore, through fractography, we investigate the impact of each effect on internal fracture.

## 2. Materials and Methods

### 2.1. NCF Composite Specimen

**Figure 1:**
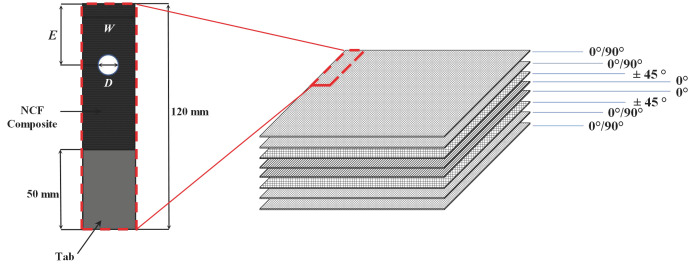
Laminate design and dimensions of the NCF composite specimen.

As shown in Figure 1, the NCF composite specimen was fabricated such that it was quasi-isotropic [(0°/90°)_2_/± 45°/0°]_S_ and possessed excellent resistance to the bearing load. Here, C-PLY BX600 NCF and BT 600 NCF (Chomarat, Le Cheylard, Auvergne Rhone Alpes, France) were used for (0°/90°) fiber orientation and ±45° fiber orientation, respectively, while PX35 carbon fiber (Zoltek, St. Louis, MO, USA) was used for 0° fiber orientation. The KFER 9400/KFCA 9410 epoxy system (Kumho P&B chemicals, Seoul, Republic of Korea) was used as a resin for fabricating the NCF specimen. The NCF composite specimen was fabricated using the infusion method. The mechanical properties of the materials that were used to fabricate the NCF composite specimen are presented in Table 1. The dimensions of the NCF specimen are presented in Table 2. The length and thickness of the specimen are 120 and 3.8 mm, respectively. The length of the grip tab for the test is 50 mm. The specimen width was set to 20 mm, and the hole diameter was machined to 5, 6, 8, and 10 mm to consider the W/D effect. Specimen cutting and holes were processed using the water-jet method.

### 2.2. Preliminary Process: Specimen Inspection and Environmental Aging

**Figure 2:**
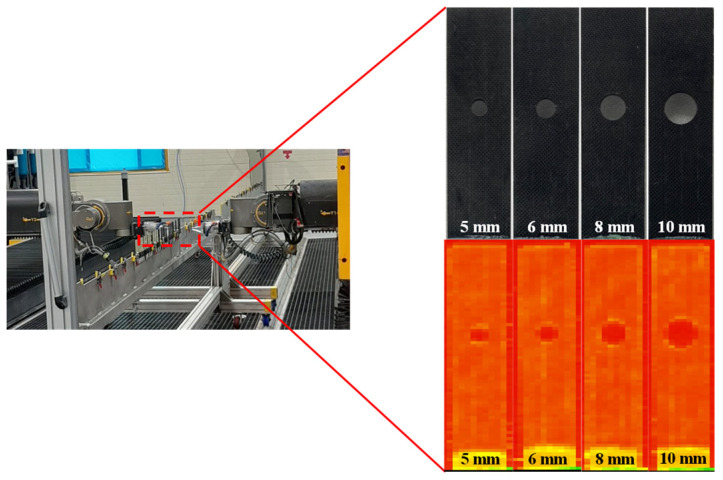
C-Scan images of NCF composite specimens.

Non-destructive testing was performed to inspect initial defects and ensure the reliability of the NCF composite specimens. The C-Scan equipment, FMS 10 Axis-Dual Squirter Ultrasonic System equipment (Marietta NDT, Marietta, GA, USA) was used. As shown in Figure 2, no defects were observed in the specimen in the C-Scan test.

To investigate the impact of the marine environment, the NCF composite specimens were aged for 500 h in saltwater and UV environments, respectively. The saltwater environment was implemented using salt fog, and this process, aging, was executed according to ASTM B117 standard [29]. The chamber interior temperature was maintained at 35 °C. The saltwater concentration was 5% with a pH of 6.5–7.2, and a saltwater spray was applied to the specimen at 1.5 mL/h. The UV environment was implemented through a xenon arc, and the specimen was aged according to ISO 4892-2 standard [30]. For the UV environment, the light source is a 6500-W xenon arc and the irradiance was 0.51 W/m^2^. The black-standard thermometer temperature was 65 °C, and the relative humidity was 50%. The rate of weight change in the specimen that was exposed to salt fog and xenon arc was compared to that of the specimen weighed before aging to investigate the impact of the marine environment on the specimen; the weight change rate is shown in Table 3.

Furthermore, to assess the applicability of the NCF composite to the ship and marine structure operating in polar environments, a tensile test was performed in a low-temperature environment using the 3119-600 series environmental chamber (Instron, Norwood, MA, USA). The temperature inside the chamber was set to −50 °C using liquefied nitrogen (LN2). Because the temperature inside the chamber (−50 °C) is not directly transferred to the NCF specimen, the tensile test was performed only after the specimen temperature reached −50 °C using a non-contact type thermometer after a duration of 30 min.

### 2.3. Mechanical Test

**Figure 3:**
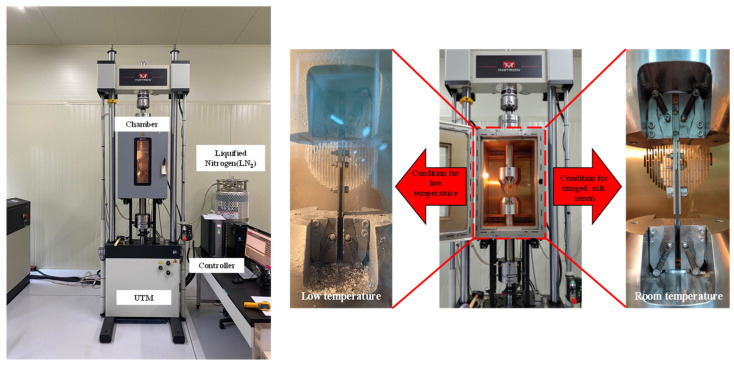
Test machine and experimental setup.

The tensile test was performed to investigate the static bearing strength of the NCF composite material. The NCF specimen was fastened to a double-lap stainless steel fixture using a bolt. For adequate fastening of the specimen during testing, a fastening torque of 15 N∙m was applied. The torque was measured three times for each specimen using a digital torque wrench to apply accurate fastening torque. As shown in Figure 3, the tensile test for mechanically fastened specimens was conducted using the 8802 servo hydraulic static and dynamic system (Instron, Norwood, MA, USA), and the load cell capacity was 250 kN. The tensile test was performed according to ASTM D5961 [31]. The tensile speed was set to 2 mm/min for testing conditions, and the test stop condition was set to 70% of the maximum load.

### 2.4. Fractography

In addition to the static bearing strength of the NCF composites, the failure mode should be analyzed. Therefore, we analyzed the internal failure of a matrix and fiber after a bolt-induced fracture. The specimen was cut using the water-jet method to facilitate the observation of the cross section between the bolt and contact part of the specimen. The cross section after cutting was investigated using a stereoscopic microscope, SZX7 (Olympus, Tokyo, Japan).

## 3. Result and Discussion

- Stress concentration and distribution

**Figure 4:**
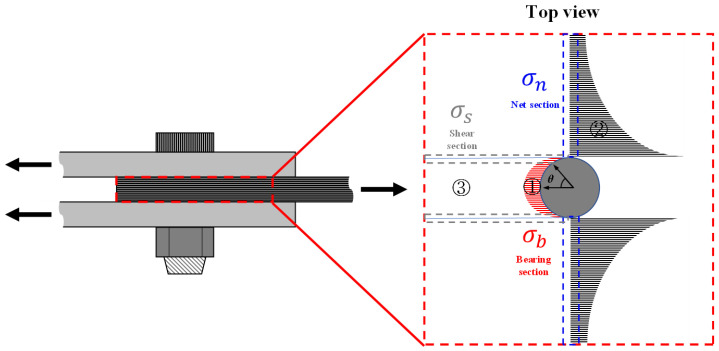
Stress distribution on the mechanically fastened part.

As shown in Figure 4, the stress concentration due to contact with a fastener and that caused by the hole notch is present in a mechanically fastened part. The concentrated stress present in the contact part between the fastener and composite material is the bearing stress. The concentrated bearing stress has radial stress with a cosine distribution, as shown in ①, and as a result, the stress concentration is caused by an uneven stress distribution according to the contact angle (*θ*). The largest value is observed when the contact angle matches the load direction. The distribution of the concentrated stress caused by the hole notch is shown in ②; the discontinuity of the structure due to the hole notch is the root cause of the stress concentration. The hole-notch-induced concentrated stress is in the opposite direction of the bearing load in the net section, and it possesses the highest value in the hole notch tip. The extent of stress concentration in ② decreases as the hole diameter increases when the width is constant. 

In Equations (1)–(4), *P* is the bearing load [N], *d* is hole diameter [mm], *W* is the specimen width [mm], *e* is the distance from hole center to free end [mm], *t* is the specimen thickness [mm], and *θ* is the angle formed with the longitudinal center line of the specimen [°].

Three major types of stress are distributed in a mechanically fastened part. First, the bearing stress distributed on the contact face between the fastener and composite and in the bearing section can be defined, as shown in Equation (1). The bearing stress is calculated by dividing the bearing load acting on the fastened part into projected areas of the fastener and the contact part of the composite material. However, the stress distribution of the actual concentrated bearing stress varies depending on the angle formed with the load direction. Therefore, the concentrated bearing stress can be expressed, as shown in Equation (2), and it includes an angle term. Second, the net stress is distributed in the net section in the specimen width, excluding the hole diameter. A load similar to the bearing load acting on the bearing section is applied to the net section. The tensile force acts in the opposite direction to the bearing load. The net stress is defined as shown in Equation (3), wherein the bearing load is divided by the cross-sectional area of the net section. Lastly, area ③ is pushed back by a fastener, and the shear stress applied in the plane is distributed in the shear section. The shear stress, which is defined in Equation (4), is calculated by dividing the bearing load of two cross-sectional areas consisting of the product of the distance from the hole center to the free end (*e*) and the specimen thickness.

(1) $$\sigma_{b}=\frac{P}{dt}$$

(2) $$\sigma_{b}=\frac{4P}{\pi dt}cos\theta$$

(3) $$\sigma_{n}=\frac{P}{(W-d)t}$$

(4) $$\sigma_{s}=\frac{P}{2et}$$

- Failure modes

**Figure 5:**
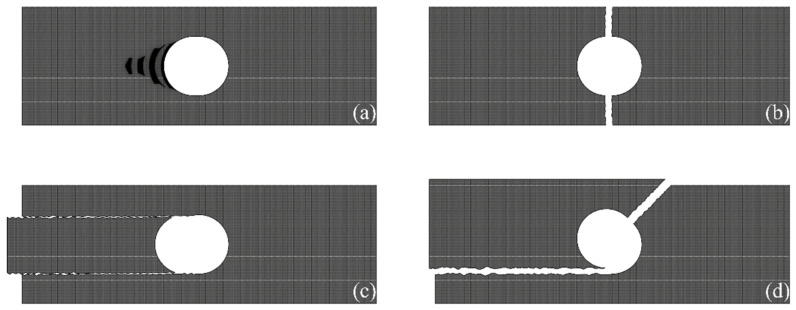
Failure modes of mechanically fastened composite material: (**a**) bearing failure mode; (**b**) net-tension failure mode; (**c**) shear-out failure mode; (**d**) cleavage failure mode.

The failure modes of mechanically fastened composite material specified in ASTM D5961 [31] are broadly categorized into four shapes, as shown in Figure 5. 

The bearing failure occurs when the bearing stress exceeds the ultimate bearing strength. Subsequently, crack assembly occurs and local failures are found in the bearing section. Bearing failure also occurs when the tensile strength of the net section is sufficient and the compressive strength of the fiber in area ③ in Figure 4 is lower than the shear strength of the shear section; it may be also seen in cases where the bearing strength is insufficient because of the high-stress concentration. 

Net-tension failure occurs when the tensile strength of the net section is lower than the bearing and shear strengths. Net-tension failure occurs when the tensile strength of the net section fiber is insufficient or due to a lack of 0°-oriented fiber supporting the major longitudinal loads. 

Shear-out failure occurs in the shear section wherein the entire area ③ in Figure 4 is pushed while maintaining the shape. Such a failure occurs because of a lack of 90° and 45° oriented fibers that prevent the detachment of area ③ or when shear strength is less than the tensile strength and bearing strength of the net section. 

The cleavage failure is a complex failure mode of shear-out failure and net-tension failure, and in such failures, longitudinal and widthwise failures occur simultaneously. Any longitudinal failure is classified as cleavage failure. Cleavage failure is caused by a longitudinal crack that is generated in the shear section at one hole tip due to a stress concentration acting on the hole notch tip, as shown in area ② in Figure 4. The stress is asymmetrically distributed in the net section due to cracks, and the net-section failure occurs in the opposite direction to the longitudinal crack propagation because of the generated moment.

### 3.1. Bearing Strength Analysis

#### 3.1.1. Width-to-Diameter (W/D) Effect on the Bearing Behavior of NCF Composites

**Figure 6:**
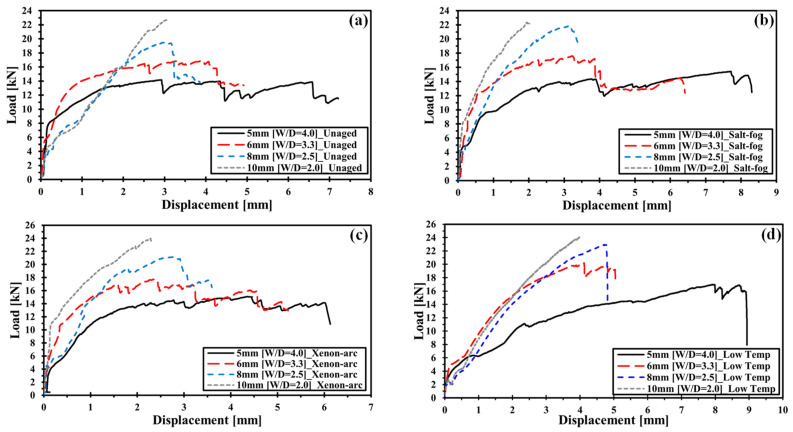
Load–displacement curve of mechanically fastened NCF composites: (**a**) load–displacement curve of unaged specimens; (**b**) load–displacement curve of salt-fog-aged specimens; (**c**) load–displacement curve xenon-arc-aged specimens; (**d**) load–displacement curve of specimens at low temperature.

This section provides a detailed comparison of the ultimate bearing load (UBL) according to the hole diameter of the NCF specimen exposed to the same environmental conditions for analyzing the W/D effect on mechanically fastened parts of carbon fiber NCF composites. Figure 6a–d show the load–displacement curve in terms of W/D in the same environment. The experimental results showed that a high UBL is observed in all specimens when the hole diameter increases due to the W/D effect. The common tendency in the entire graph is that UBL moves towards the upper-left side as W/D decreases, regardless of the exposed environment. Here, UBL increases as W/D decreases mainly because local stress decreases due to mitigation of stress concentration, and larger loads are required to induce failure of a material. The reduction in failure displacement may be attributed to the reduction in the local crack assembly of the contact part caused by the mitigation of stress concentration [6]. It was confirmed that the W/D effect mainly determines the bearing strength from the comparison of the load–displacement curve with respect to W/D in the same environment. The environmental effect did not have a significant impact on the overall tendency of the increase in UBL as W/D decreases. However, it may affect the determination of the UBL value.

#### 3.1.2. Environmental Effect on the Bearing Behavior of NCF Composites

**Figure 7:**
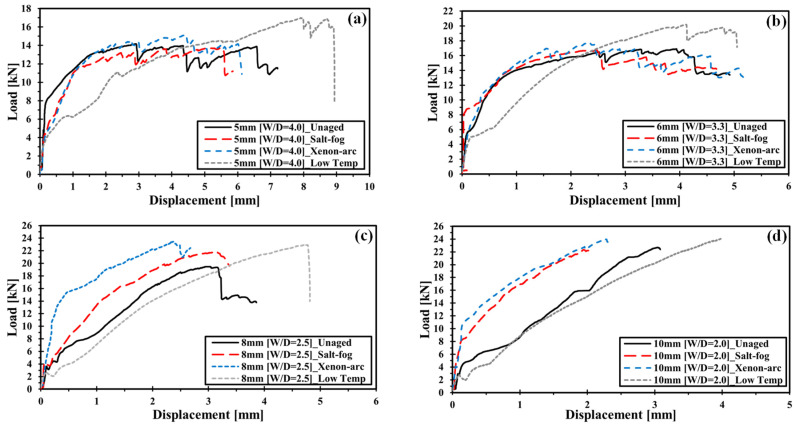
Load–displacement curve of mechanically fastened NCF composites: (**a**) load–displacement curve of diverse conditions for a 5 mm hole diameter; (**b**) load–displacement curve of diverse conditions for a 6 mm hole diameter; (**c**) load–displacement curve of diverse conditions for an 8 mm hole diameter; (**d**) load–displacement curve of diverse conditions for a 10 mm hole diameter.

In this section, the UBL of the specimens with the same hole diameter was compared to those exposed to different environmental conditions to analyze the environmental effect on mechanically fastened parts of carbon fiber NCF composites. Figure 7a–d show the load–displacement curve according to the environment in the same W/D. 

The experimental results showed that compared to unaged specimens, the UBL of the specimen that was exposed to low temperature and xenon arc increased for all hole diameters. The specimen that was exposed to low temperatures demonstrated the most significant increase in UBL. 

The increase in UBL due to a low temperature may be attributed to the decrease in molecular mobility of epoxy resin [32]. In addition, the interface strength increases because of the compressive stress between the fiber and resin, which is induced by the traversal expansion of carbon fiber and contraction of resin in low temperatures [33,34,35]. The increase in UBL due to xenon arc is induced by the resin-hardening effect [19].

The specimens that are exposed to low temperatures exhibit relatively low stiffness compared to those exposed to other environments in nonlinear behaviors regardless of W/D. Further, the specimens exposed to low temperatures have higher failure displacement values compared to those exposed to other environments. Microcracking may easily occur in epoxy resin in cases where there is an increase in brittleness due to a low-temperature environment. Crack assembly is accelerated when a stress concentration occurs. However, the tendency of low stiffness is similar to the result of the unaged specimen with an increase in hole diameter. This may be attributed to the fact that crack assembly occurs less frequently as the stress concentration is mitigated with the increase in hole diameter.

The specimen that is exposed to salt fog and xenon arc shows a similar stiffness level to that of the unaged specimen in terms of nonlinear behavior when the hole diameter is 5 and 6 mm. However, the stiffness is high when the hole diameter is 8 and 10 mm. The effects of salt fog and xenon arc on stiffness are related to the occurrence of resin hardening due to marine environments. No significant effect is found because xenon light or salt spray cannot easily penetrate the specimens due to a small hole notch when the hole diameter is 5 or 6 mm, whereas penetration is easy when the hole diameter is 8 or 10 mm. The reasons for this are presented in Table 3, which also indicates that the weight change rate for hole diameters of 8 and 10 mm is greater than that for hole diameters 5 and 6 mm. It can be inferred that the stiffness increases with the occurrence of resin hardening due to environmental effects.

#### 3.1.3. Comparison of the Bearing Strength

**Figure 8:**
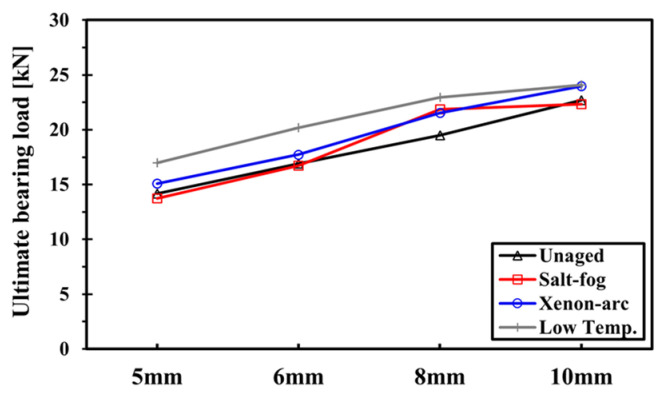
Comparison of the ultimate bearing load.

From Figure 8, it can be seen that UBL is the highest in the specimen that is exposed to low temperatures, and it is the second highest in the specimen exposed to xenon arc. Further, similar results are observed in the salt fog and unaged specimens. The specimen exposed to salt fog shows a high value only when the hole diameter is 8 mm. Regardless of the environments in which they are exposed, all specimens witnessed an increase in UBL with the hole diameter. This further implies that the W/D effect is a dominant factor when determining UBL tendency rather than the environmental effect. 

The margin of increase in UBL of the low-temperature and unaged specimen is gradual, while the W/D effect is more dominant. In contrast, the specimens exposed to salt fog and xenon arc witnessed a considerable increase in UBL when the hole diameter was in the range of 6–8 mm, and the largest increase was found in the salt fog specimen. This may be attributed to the resin hardening caused by the marine environmental effect, which is primarily concentrated on the specimen surface and notch. 

From Table 3, it can be seen that the rate of weight change in the salt fog and xenon arc specimen is similar for 5–6 mm and 8–10 mm, respectively. It was confirmed that there was a considerable rise in the saltwater absorption rate in the 6–8 mm range of hole diameter in the specimen exposed to salt fog. Also, it was confirmed that there was an increase in dehydration in the 6–8 mm range of hole diameter in the specimen exposed to xenon arc.

The impact on UBL determination may vary depending on the location of resin hardening caused by salt fog and xenon arc applied to the specimens. The specimens were laminated at [(0°/90°)_2_/± 45°/0°]_S_, wherein the 0°-oriented fiber of surface layers mostly supports the bearing load, which is the 0°-oriented in-plane load. The resin hardening due to the xenon arc mostly occurred in the 0°-oriented fiber layers, which are the surface layers of the specimen. Therefore, the resin hardening in the 0°-oriented fiber layers of surface layers has a significant impact on the determination of UBL. In particular, the salt fog witnesses a higher margin of increase compared to the xenon arc due to the following reasons. In the specimen exposed to the salt fog environment, saltwater penetration occurred in core layers, including surface layers, through a hole notch for 500 h. Because this phenomenon induces resin hardening over the entire core layers as well as the surface layers, the largest increase in UBL is observed in the 6–8 mm range where saltwater absorption increases with the hole notch diameter.

#### 3.1.4. Energy Absorption Capacity of NCF Composites

Toughness is a measure for expressing the extent of energy absorption of material before fracture due to external forces. The toughness coefficient is expressed as the area under the stress–strain curve. In general, when converting a load–displacement curve into a stress–strain curve, it is calculated by dividing the load dimension by the [L^2^] scale area and by dividing the displacement dimension by the [L^1^] scale area. According to Equation (1), the bearing stress exhibits irregularity when converting load values to stress values mainly because the value of the term representing the areas in the denominator varies according to the hole diameter. For example, as shown in Figure 6a, the UBL of a 5 mm hole diameter for the unaged specimen is greater than that for the 10 mm hole diameter by a factor of 1.6. During the conversion of the UBL to the ultimate bearing strength (UBS), it is observed that the UBS of the specimen with a hole diameter of 10 mm is smaller because the value divided by the denominator of Equation (1) is twice as large as that of the 5 mm specimen. 

The presence of irregularities in the conversion process is because we compared the energy absorption capacity of the specimens that underwent the W/D and environmental effects by using the load–displacement curve. The area under the graph of a load–displacement curve is the product of the load dimension [M^1^L^1^T^−2^] and displacement dimension [L^1^], and thus, it possesses the [M^1^L^2^T^−2^] dimension. This is identical to the energy dimension [M^1^L^2^T^−2^], and it signifies the fracture energy, which is the amount of energy absorbed until a material fractures.

**Figure 9:**
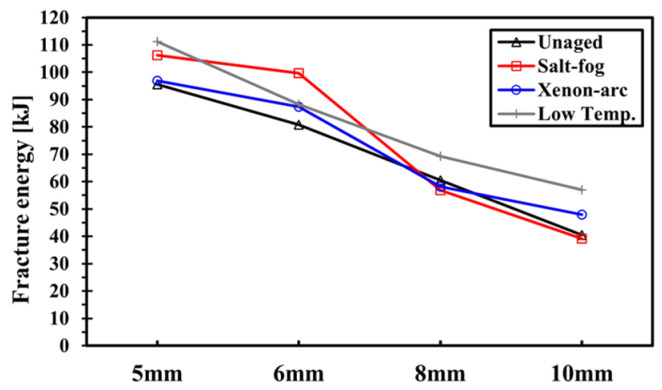
Comparison of the fracture energy for diverse environments.

Figure 9 shows the graphs of the fracture energy in Table 4. The fracture energy decreases as the hole diameter increases, thus demonstrating a contrasting tendency from the UBL. Fracture energy is the highest in the specimen that is exposed to low temperatures. On the contrary, the unaged specimen and the specimens exposed to salt fog and xenon arc showed a similar fracture energy.

It is worth noting that the salt fog and xenon arc specimens in Figure 8 witnessed a dramatic increase in UBL in the diameter hole range of 6–8 mm, and a drastic decrease is observed in Figure 9. A sharper slope of the salt fog sample is observed in the UBL compared to that of the xenon arc sample. Similar to the increasing tendency for UBL, the low-temperature and unaged specimens witnessed a gradual decrease. The main reason is that the specimen exposed to xenon arc exhibits local resin hardening in the surface layers, and this effect was transmitted to the core layers including the surface layers in the salt fog specimen.

These experimental results suggest that the specimen exposed to low temperatures requires higher fracture energy than the unaged, salt fog, and xenon arc specimens due to the increased mechanical properties of the materials at low temperatures. Therefore, the carbon fiber NCF composite is suitable for use in marine structures and polar sailing ships mainly because of its excellent strength and energy absorption capacity.

### 3.2. Failure Mode Analysis

#### 3.2.1. Observation of Damage and Failure Mode

**Figure 10:**
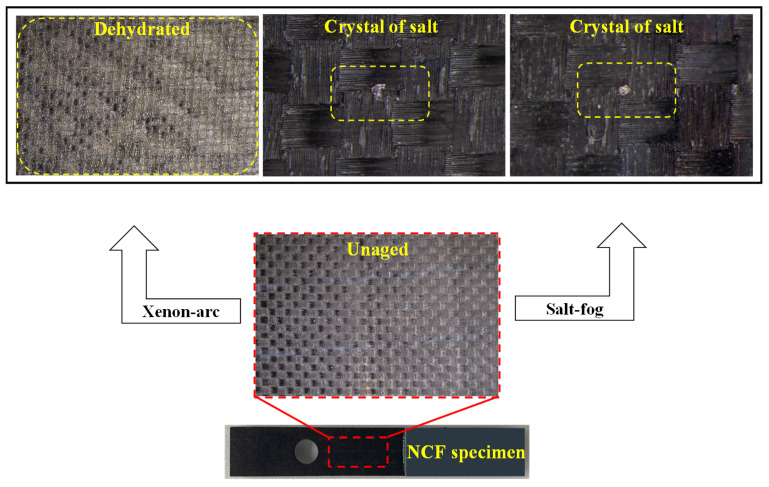
Surface changes in NCF specimens after aging in marine environments.

Figure 10 shows a comparison of the unaged specimen and the specimens that have been aged for 500 h in salt fog and xenon arc before conducting the static bearing test. A crystal of salt was observed on the specimen that was exposed to salt fog. Besides the two types of changes on the surface, other kinds of damage such as delamination or swells were not found. Dehydration occurred on the surface of the specimen exposed to xenon arc. Contrary to the unaged specimen, a bumpy surface was observed due to the hardening of the epoxy resin on the surface, in addition to yellowing. In contrast, except for dehydration, no other changes were observed.

**Figure 11:**
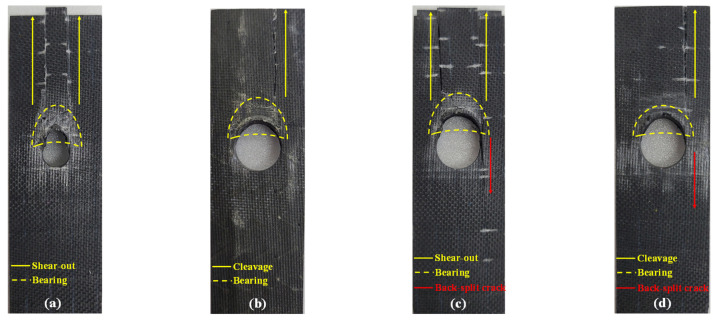
Failure modes of mechanically fastened NCF composites: (**a**) shear-out and bearing failure; (**b**) cleavage and bearing failure; (**c**) shear-out, bearing, and back-split crack failure; (**d**) cleavage, bearing, and back-split crack failure.

Figure 11 illustrates the failure modes generated in the carbon fiber NCF composite specimens. Four types of failure modes including bearing, shear-out, cleavage, and back-split cracks were observed.

The bearing failure was found in all specimens. However, the degree of bearing failure was mitigated as the hole diameter increased. The bearing failure may be attributed to the accumulated local failure caused by the crack assembly, and it occurred in the contact part due to the concentrated bearing stress acting on the bearing section. The scale of the bearing failure tends to decrease as the hole diameter increases mainly because the crack assembly is mitigated as the stress concentration is reduced. The causes of shear-out and cleavage failure are related to the laminate design of the specimen. The specimen is laminated as [(0°/90°)_2_/± 45°/0°]_S_ wherein the 0°-oriented fiber accounts for a large portion. The specimen has a high longitudinal tensile and compressive strength due to the large portion of the 0°-oriented fiber that supports longitudinal loading. However, the transverse and shear strengths are insufficient. Along with the 0°-oriented fiber, stress concentration on the hole notch tip accelerates the generation of longitudinal cracks. Therefore, shear-out and cleavage failure are more likely to occur. The induced shear stress caused by the strain difference between the opposite part of the bearing load from the hole notch and the net section is the root cause of back-split cracks [6]. A large strain occurs in the net section where the same level of tensile load as the bearing load is applied, while a small strain occurs in the back part of the hole notch, thus inducing shear stress due to the strain difference.

**Figure 12:**
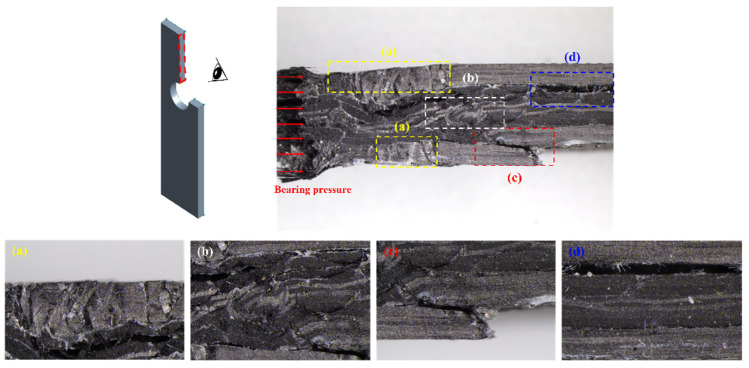
Various failures on the cross section of a mechanically fastened NCF composite specimen: (**a**) fiber collapse, (**b**) fiber buckling, (**c**) fiber kinking, (**d**) delamination.

Figure 12 shows a microscope image of the cross section of a damaged specimen after performing the static bearing test. Various types of failures are observed within the specimen, as shown by regions (a)–(d) such as fiber collapse, fiber buckling, fiber kinking, and delamination. Here, (a) shows fiber collapse and (c) shows that fiber kinking occurred in the (0°/90°) layer, which are complex failures of the in-plane shear mode of the 0°-oriented fiber and tensile opening mode of the 90°-oriented fiber. Fiber buckling in (b) is the result of the 0°-oriented fiber that primarily supports the bearing load being damaged by the in-plane load. Delamination in (d) occurred between the (0°/90°) layer and ±45° layer due to the strain difference between the two layers. 

#### 3.2.2. Analysis of W/D and Environmental Effects on the Failure Mode 

Table 5 summarizes the failure modes that are observed in the carbon fiber NCF composite specimens. For a comparison of the W/D and environmental effects on the determination of failure modes, the failure mode of the unaged specimen was used as the reference. The failure mode of the unaged specimen is not affected by the environment and is determined solely based on the W/D effect. When the impact of W/D on the determination of the failure modes was analyzed, the shear-out and bearing failures were the major failure modes in the 3.3 ≤ W/D ≤ 4.0 section. In the W/D = 2.5 section, a combination of both the cleavage and shear-out failures were observed, and bearing failure was commonly observed. In the W/D = 2.0 section, cleavage and bearing failures were dominant. From the failure mode distribution results, it was confirmed that the determination of failure mode due to only W/D effects occurs in the W/D = 2.5 section, which is the transition section, and the shear-out failure is converted to cleavage failure as W/D decreases. The specimens that were exposed to salt fog, xenon arc, and low temperatures showed a similar tendency as the unaged specimens. The specimen that was exposed to salt fog showed the same failure mode as the unaged specimen. However, the changes in the transition section and new failure mode were observed in the xenon arc and low-temperature specimens.

Compared to the unaged specimen, cleavage failure was observed in the early stages at W/D = 3.3 instead of W/D = 2.5 in the specimen that was exposed to xenon arc. While the failure mode transition section is at W/D = 2.5 in the unaged specimen, a mixture of shear-out and cleavage failures was observed at W/D = 2.0 in the specimen exposed to xenon arc. Therefore, the failure mode transition section of the xenon arc specimen is 2.0 ≤ W/D ≤ 3.3, which is the widest. A back-split crack was observed in the specimen that was exposed to xenon arc at W/D = 2.0, which was not observed in the specimen exposed to salt fog and the unaged specimen. 

In the specimen that was exposed to low temperature, a mixture of shear-out and cleavage failures was observed at W/D = 2.0 and in the transition section of the unaged specimen including W/D = 2.5. Therefore, the failure mode transition section of the specimen exposed to a low temperature is 2.0 ≤ W/D ≤ 2.5, which is longer than the transition section of the unaged specimen. Similar to the specimen exposed to xenon arc, the specimen exposed to a low temperature exhibited a back-split crack at W/D = 2.0.

A back-split crack specifically occurred when the diameter was 10 mm in the specimens exposed to xenon arc and a low temperature. As mentioned previously, the back-split crack is caused by the induced shear stress resulting from the strain difference between the back part of the hole notch and net section. The specimens exposed to xenon arc and a low temperature have the highest UBL values when the hole diameter is 10 mm, as shown in Figure 8. Therefore, a large strain is generated due to the high reactive force of UBL acting on the net section.

**Figure 13:**
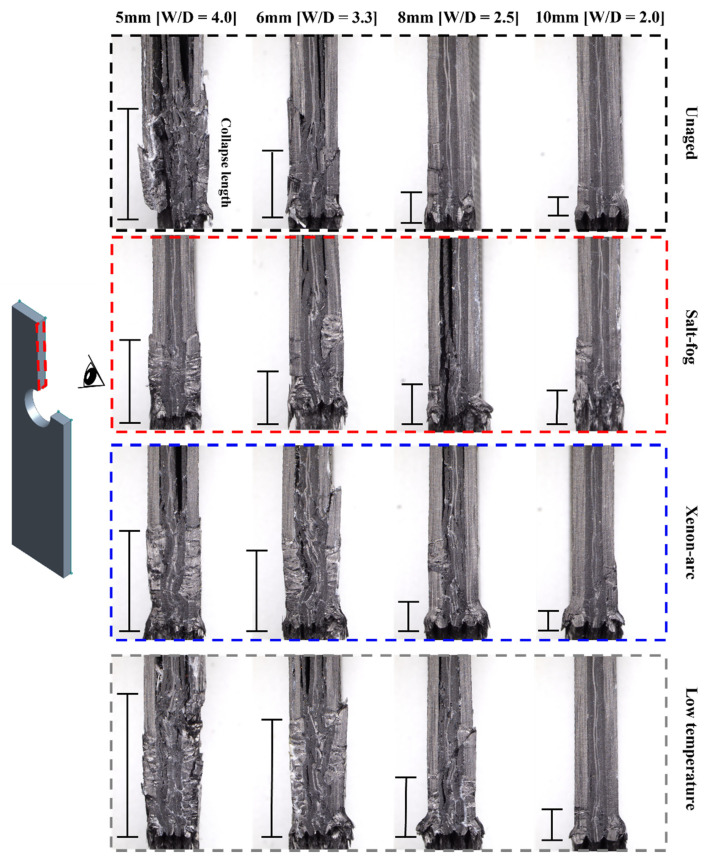
Failure on the cross section of mechanically fastened NCF composite specimens.

Figure 13 shows a microscope image of the cross section of the specimen that experienced failure after the static bearing test. The W/D effect is demonstrated through the collapsed length of the cross section. The collapse length tends to decrease as W/D decreases. This implies that crack assembly is mitigated as the stress concentration is reduced when W/D decreases. The proportional relationship between W/D and the collapse length is identical irrespective of the exposed environment. 

In the unaged specimen, fiber collapse, fiber buckling, fiber kinking, and delamination were observed when the hole diameter was 5 and 6 mm. The unaged specimen witnessed relatively less damage in core layers as the hole diameter increased, and delamination did not occur in the specimens with hole diameters of 8 and 10 mm.

In the specimen that was exposed to salt fog, fiber collapse, fiber buckling, and fiber kinking were primarily observed when the hole diameter was 5 and 6 mm, while delamination of the core layers was observed when the hole diameter was 6 and 8 mm. The degree of failure when the hole diameter was 5 and 6 mm was similar to that of the unaged specimen. In contrast, the failure in the core layers was more serious compared to the unaged specimen when the hole diameter was 8 and 10 mm, and this may be attributed to the fact that saltwater can easily penetrate the core layers as the hole diameter increases. 

In the specimen that was exposed to xenon arc, fiber collapse, fiber buckling, fiber kinking, and delamination were observed when the hole diameter was 5 and 6 mm, and the degree of damage was similar to that of the unaged specimen. Fiber kinking and delamination occurred when the hole diameter was 8 and 10 mm, and the degree of damage in core layers was less than that of the salt fog specimen. The main reason for these results is that xenon arc has a greater impact on surface layers, and therefore, the increase in hole diameter and the effect of xenon arc on core layers are not significantly correlated. 

In the specimen that was exposed to a low temperature, fiber collapse, fiber buckling, fiber kinking, and delamination were observed when the hole diameter was 5, 6, and 8 mm. A more severe fiber collapse occurred compared to the unaged specimen mainly due to the brittle behavior of epoxy resin at a low temperature. Micro cracking is more likely to occur as the brittleness of the epoxy resin increases, and fiber collapse also occurs frequently due to the accelerated fracture of the resin layer supporting the fiber.

## 4. Conclusions

We conducted experiments on W/D and environmental effects, which have a considerable impact on the determination of the failure modes and the bearing strength of the mechanically fastened parts in carbon fiber NCF composites. 

The W/D effect is significant for determining the bearing strength. A decrease in W/D results in an increase in the bearing strength. Contrarily, the fracture energy tends to decrease as W/D decreases. Consequently, the W/D effect has a greater impact than the environmental effect when determining the bearing strength of the mechanically fastened NCF composites. Similarly, the W/D effect has a significant impact when determining the failure modes. In the unaged specimen, it was confirmed that W/D = 2.5 is the transition section where shear-out failure converts to cleavage failure. 

The bearing strength of the NCF composite specimen, which was aged for 500 h in salt fog, is similar to or higher than that of the unaged specimen. Furthermore, the fracture energy is also slightly higher than or similar to the results of the unaged specimen; therefore, aging for 500 h in salt fog does not have a significant impact on the mechanical performance of mechanically fastened carbon fiber NCF composites. Failure modes identical to the unaged specimen are observed, and there were no changes in the failure mode transition section in a saltwater environment. 

The bearing strength of the carbon fiber NCF specimen aged for 500 h in xenon arc is greater than that of the unaged specimen by 5%. The reason for the increased bearing strength of the specimen exposed to xenon arc is resin hardening. Further, the xenon arc primarily had a local impact on the surface layers of the specimen. However, the failure mode was changed after passing through the widest transition section of 2.0 ≤ W/D ≤ 3.3, unlike in the unaged specimen, which experiences the failure mode transition section at W/D = 2.5. Therefore, 500 h of exposure to xenon arc can directly affect the failure mode changes in the mechanically fastened parts in carbon fiber NCF composites. 

The bearing strength of the carbon fiber NCF specimen exposed to a low temperature of −50 °C was higher than that of the unaged specimen by 10%. The cause of this increase in bearing strength in the low-temperature specimen was the brittle behavior of epoxy due to the temperature decrease. The failure mode was changed after passing through a wider transition section of 2.0 ≤ W/D ≤ 2.5, unlike in the unaged specimen, which experienced the failure mode transition section at W/D = 2.5. Therefore, the low-temperature environment can directly affect the failure mode changes in the mechanically fastened parts in carbon fiber NCF composites.

Our findings demonstrate that the carbon fiber NCF composite is an appropriate material for ships and marine structures. When the specimen is exposed to a marine environment such as salt fog and xenon arc for 500 h, the specimen witnesses an increase in the bearing strength and energy absorption capacity compared to the unaged specimen. The bearing strength and energy absorption capacity of the specimen exposed to a low-temperature environment are superior to those of the unaged specimen, and they can therefore be integrated into structures that are primarily operated in polar environments.

Future research can be conducted to evaluate long-term exposure to marine environments, the impact of a mixture of marine environments and salt fog aging under polar environments and a comparison with other composites. 

## Figures and Tables

**Table 1:** Constituent material properties.

Material Properties		
Fiber orientation	0°, ±45°	0°/90°
Fiber product	PX 35	TRW 40
Tensile strength [MPa]	4137	4340
Tensile modulus [GPa]	242	235
Elongation [%]	1.7	1.8
Density [g/cc]	1.81	1.85

**Table 2:** Dimensions of the NCF composite specimens.

D (mm)	W (mm)	E (mm)	W/D	E/D	t (mm)
5	20	30	4.0	6.0	3.8
6	20	30	3.3	5.0	
8	20	30	2.5	3.8	
10	20	30	2.0	3.0	

D: Diameter of hole; W: Width of specimen; E: Distance of hole center to free edge; t: Thickness of specimen.

**Table 3:** Weight change rate.

Environments	Average Weight Change Rate [%]			
	5 mm	6 mm	8 mm	10 mm
Salt fog	0.66	0.62	0.96	0.97
Xenon arc	−0.36	−0.31	−0.43	−0.41

**Table 4:** Fracture energy of NCF composites.

Environment	Fracture Energy [J]			
	5 mm	6 mm	8 mm	10 mm
Unaged	95.57	80.75	60.53	40.52
Salt fog	106.24	99.67	56.78	39.09
Xenon arc	96.86	87.39	58.18	47.99
Low temperature	111.15	88.26	69.19	56.96

**Table 5:** Failure modes of NCF composites.

Environments	Hole Diameter [W/D Ratio]			
Unaged	5 mm [W/D = 4.0]	6 mm [W/D = 3.3]	8 mm [W/D = 2.5]	10 mm [W/D = 2.0]
#1	S+B	S+B	S+B	C+B
#2	S+B	S+B	S+B	C+B
#3	S+B	S+B	C+B	C+B
Salt fog	5 mm [W/D = 4.0]	6 mm [W/D = 3.3]	8 mm [W/D = 2.5]	10 mm [W/D = 2.0]
#1	S+B	S+B	C+B	C+B
#2	S+B	S+B	S+B	C+B
#3	S+B	S+B	C+B	C+B
Xenon arc	5 mm [W/D = 4.0]	6 mm [W/D = 3.3]	8 mm [W/D = 2.5]	10 mm [W/D = 2.0]
#1	S+B	C+B	S+B	S+B
#2	S+B	C+B	C+B	C+B
#3	S+B	S+B	C+B	C+B+BS
Low temperature	5 mm [W/D = 4.0]	6 mm [W/D = 3.3]	8 mm [W/D = 2.5]	10 mm [W/D = 2.0]
#1	S+B	S+B	C+B	C+B
#2	S+B	S+B	S+B	S+B
#3	S+B	S+B	C+B	S+B+BS

B: Bearing failure; C: Cleavage failure; S: Shear-out failure; BS: Back-split crack failure.

## Data Availability

The original contributions presented in the study are included in the article, further inquiries can be directed to the corresponding author.
